# Upper Extremity Transplant Rehabilitation Protocol

**DOI:** 10.1055/s-0045-1810010

**Published:** 2025-08-20

**Authors:** Ravi Sankaran, Subramaniya Iyer, Mohit Sharma, Jimmy Mathew, Sam Thomas

**Affiliations:** 1 Department of Physical Medicine and Rehabilitation, Amrita Hospital, Kochi, Kerala, India; 2 Department of Plastic Surgery, Amrita Hospital, Kochi, Kerala, India; 3 Department of Plastic Surgery, Amrita Hospital, Faridabad, Haryana, India

**Keywords:** hand transplantation, rehabilitation, functional recovery, motor re-education, occupational therapy, nerve regeneration

## Abstract

**Background:**

Multilevel upper extremity transplant presents unique rehabilitation challenges due to the complexity of restoring function and integration across multiple joints and tissue types.

**Materials and Methods:**

This article outlines the development and implementation of a pioneering rehabilitation protocol designed specifically for recipients of upper extremity transplant.

**Results:**

The rehabilitation protocol was structured in four progressive phases, emphasizing early preservation, neuromuscular re-education, activities of daily living retraining, and return to society.

**Discussion:**

The initial phase focuses on correct patient selection and preparation, wound healing, edema management, and maintaining passive range of motion while protecting the vascular anastomoses. As healing progresses, a tailored exercise regimen incorporating mirror therapy, task-oriented activities, and proprioceptive training is introduced to facilitate cortical remapping and sensory recovery.

**Conclusion:**

Multidisciplinary collaboration, including physiatrists and occupational or physical therapists, is crucial in addressing their recovery.

## Introduction

The first successful hand transplant was done in 1999. 1 Since then, the approximate number of registered transplants done to date is approximately 200+ across 50+ centers. The first in Southeast Asia was done in India in 2015. 2 Many regional centers have emerged. Currently, hand transplant is accepted as a worldwide standard reconstructive option. While the surgery is technically challenging, gaining function thereafter is critical. Although few protocols exist, none addresses each level of transplant. 3 4 The closest model is for upper extremity replant. This is the first multilevel rehabilitation protocol for upper extremity transplantation. It details presurgical preparation, rehabilitation, and return-to-society care. This protocol was made after 34 hand transplants in 18 patients, the current largest series in the world.

## Materials and Methods

There are four stages in the rehabilitation of an upper limb transplant recipient. They are the stages of preparation, anticipated movements, aggressive rehabilitation, and the maintenance phase. The foundation for good outcomes is the right patient, right time, right rehabilitation. Rehabilitation, being patient-specific and highly variable, rarely conforms to a structured plan. Complications can emerge. Rapid gains followed by plateaus in performance may also be noted. Without mapping the trajectory along an expected path, overreaction to apparent loss or complacence with lack of gains may result. The subsequent information provides a structured framework for assessment, goal setting, and appropriate interventions.

### Stages of Preparation

An initial assessment considers the following: pain, range of motion (RoM) of existing joints, power of existing muscles, comorbidities that can affect rehabilitation, psychological evaluation, vocational assessment and capacity, educational status, social support, and understanding of what the rest of their life will be like ( Table 1 ).

**Table 1:** Summary of stages of preparation

Phase/duration/name	Goal	Interventions
0/indefinite/prehabilitation	Keep residual limbs supple and ready for surgery Maintain muscle bulk/prevent disuse atrophy	Maintenance therapy: ask recipients to keep contracting the existing muscles of the amputated extremities, develop all existing upper body muscles and core muscles
1/days 0–30/immediate post-transplant	Limb protection, preactivity, body mobilization, vitals, nutrition	Splints, protection, and early passive and active mobilizations (for distal hand transplants), locomotion (with a gutter walker)

The optimal recipient for transplant is 2 years from the event otherwise without comorbidities, bilateral amputation, has tried all other rehabilitation measures (including appropriate prosthetics), and is highly motivated. 5 6 The following are basic assessments.

*Exercise and activity* : Enquire into sleep adequacy. Identify and reduce harmful behaviors. Ensure patients walk 8,000 steps per day. Optimize the body mass index with a whole food plant-based diet. Ensure they have a good social support structure. Resistance band exercises tone up the core and functional muscles in the residual limb. Educational level assessment and vocational surveys should be done. Often, factors like this are overlooked and can lead to complications later. Implementing the preoperative exercises is preparation for what follows.

*Social support network* : A strong and supportive social network, including the transplant team, family, and friends, plays a vital role in the rehabilitation of these persons. 7 Good support can ensure the recipients' compliance with immunosuppressants, active participation in rehabilitation, keeping up the hospital visits, and successful integration of the transplanted limb into their sense of self. 8 9 10 11 Social and emotional support can significantly enhance both physical recovery and psychological well-being and promote better long-term outcomes.

*Preoperative rehabilitation counseling* : It is essential to thoroughly inform and educate patients and their families about initial physical dependence of the patient. They should be made aware of the need for long-term rehabilitation, committing time every day for therapy and compliance required to achieve the best functional outcomes. Psychological evaluation and counseling should also be done. 4 12

*Mobilization* : Once the postoperative mobilization is permitted, it must be performed during the waking hours before all meals 10 times for each joint. Drains will be progressively removed as residual volumes reduce.

*Edema management* : Significant edema in the early postoperative period is expected. 13 It affects flexibility and joint mobility. Chronic edema impedes sensory and motor recovery owing to intraneural ischemia. 14 Measures to resolve edema early are important. Compression bandages and decongestive massage are needed when muscles do not voluntarily act.

*Orthoses* : Once the hands are transplanted, the patient spends the first 15 days in the intensive care unit. As the postoperative issues are handled, the patient is mobilized to stand and walk.

Distal transradial recipients are mobilized with knucklebender splints.

Proximal transradial recipients will have the same with a polypropylene (PPE) full-length cockup splint and bilateral broad arm slings.

Midarm recipients will need the same and a gutter walker with front wheels and straps to retain the limbs in the frame. The gutters need to be custom-formed using an ankle–foot orthosis mold. This will accommodate the transplanted limb girth. It should extend above the limb height and remain open above.

**Fig. 1:**
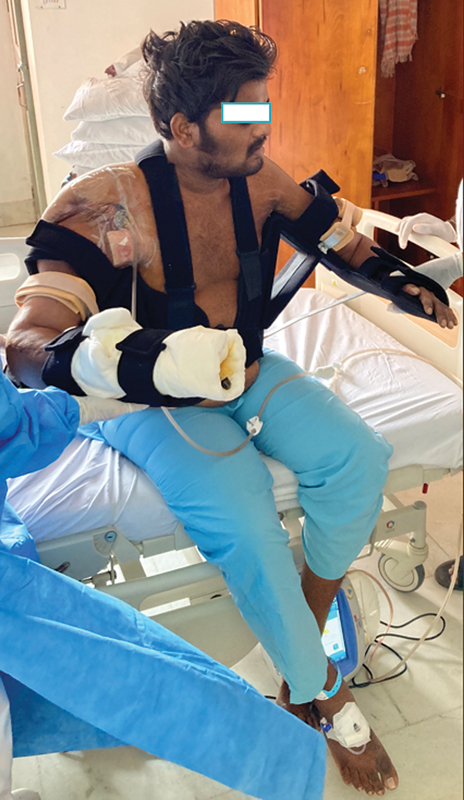
Post shoulder level transplant recipient in custom orthosis.

Shoulder-level recipients pose the greatest challenge. After a shoulder-level transplant, the arm has to be slowly lowered to the trunk over months. To mobilize the recipient from supine-to-sit and sit-to-stand requires a trunk–shoulder–elbow–wrist hand orthosis. A removable frame that can slowly bring the shoulder into adduction is needed. What we describe is a unique brace-made custom for this situation. The PPE shell will wrap around the trunk and be opened on the contralateral side of the transplant. A D-ring strap with buckles can be a closure. It should extend from 5 cm below the anterior and posterior axillary folds to the iliac crest on that side. Adequate ethylene vinyl acetate (EVA) padding will reduce discomfort on the body prominence. To this frame, a paired dial lock brace will be mounted both anteriorly and posteriorly. The proximal limbs are mounted on the PPE shell. The distal attaches to a gutter (as mentioned prior) that will sit at a 90-degree angle to the trunk. An additional central aluminum strut of 5 × 1 × 30 cm can be added to support the limb weight if needed. The gutter covers the arm segment. A PPE cockup splint positioned parallel to the trunk/perpendicular to the gutter is mounted with an aluminum strut. The whole brace is padded with EVA and has Velcro straps. The orthosis depth is enough to contain the padded limb and extend by 1 cm ( Fig. 1 ).

### Stage of Anticipated Movement

This is a critical phase of care. It has been generalized here ( Table 2 ) but is subject to individual variability. Stage naming follows a temporal progression starting from distal most to proximal (to the body's midline). The highest level of performance is the long finger flexor function. Every subsequent stage is a predecessor. The recipient should have a weekly review with the physiatrist. Progress is marked, and goals for the week are set ( Table 2 ).

**Table 2:** Summary of stage of anticipated movement

Phase/functional level/duration of the phase/distance from operative site to hypothenar NMJ	Goal	Joints and muscles: What exists (active joints) What is missing (inactive joints) What needs to be preserved (active muscles) What needs to be redeveloped (inactive muscles)	Interventions
2a/distal transradial/3 mo/10 cm	Long finger flexion begins (C8, T1)	Active joints: scapulothoracic, shoulder, elbow, RUJs Inactive joints: wrist, MCP, PIP, DIP Active muscle groups: shoulder, elbow, forearm, wrist Inactive muscles: intrinsics, thenar, hypothenar	PRoM: for inactive joints AARoM: for active joints whose muscles are MRC less than 4 ARoM: for joints whose muscles are MRC 4 PRE: for joints whose muscles are MRC 5 Functional tasks: see functional task rubric Orthotics: knucklebender Electric stimulation: for inactive muscles Practical issues: based on tendon repairs and muscle recovery, fingers may end up crossing or irritating their synovial sheaths, custom splints are of value
2b/proximal transradial/9 mo/25 cm	Wrist extension Begins (C7,8)	Active joints: scapulothoracic, shoulder, elbow Inactive joints: RUJs, wrist, MCP, PIP, DIP Active muscles groups: shoulder, elbow, forearm Inactive muscles: long finger flexors and extensors, wrist flexors and extensors, intrinsics, thenar, hypothenar	PRoM: for inactive joints AARoM: for active joints whose muscles are MRC less than 4 ARoM: for joints whose muscles are MRC 4 PRE: for joints whose muscles are MRC 5 Functional tasks: see functional task rubric Orthotics: knucklebender and cockup Electric stimulation: for inactive muscles Practical issues: the radioulnar joints may become malaligned resulting in restricted pronation and supination
2c/midarm/12 mo/35 cm	Supination/pronation Begins (C7,8) Elbow flexion begins (C6,7)	Active joints: shoulder, scapulothoracic Inactive joints: elbow, RUJs, wrist, MCP, PIP, DIP Active muscles groups: shoulder, elbow Inactive muscles: elbow flexors and extensors, supinators, pronators, long finger flexors and extensors, wrist flexors and extensors, intrinsics, thenar, hypothenar	PRoM: for inactive joints AARoM: for active joints whose muscles are MRC less than 4 ARoM: for joints whose muscles are MRC 4 PRE: for joints whose muscles are MRC 5 Functional tasks: see functional task rubric Orthotics: knucklebender and cockup with broad arm slings Electric stimulation: for inactive muscles Practical issues: delay to function creates anxiety, a low profile splint that provides dynamic wrist extension can position the fingers to act earlier
2d/below shoulder/18 mo/60 cm	Shoulder abduction Begins (C5,6)	Active joints: scapulothoracic Inactive joints: shoulder, elbow, RUJs, wrist, MCP, PIP, DIP Active muscles groups: shoulder abductors, external rotators, extensors Inactive muscles: elbow flexors and extensors, supinators, pronators, long finger flexors and extensors, wrist flexors and extensors, intrinsics, thenar, hypothenar	PRoM: for inactive joints AARoM: for active joints whose muscles are MRC less than 4 ARoM: for joints whose muscles are MRC 4 PRE: for joints whose muscles are MRC 5 Functional tasks: see functional task rubric Orthotics: knucklebender and cockup with broad arm slings after 3 mo of trunk shoulder–elbow–wrist orthosis Electric stimulation: for inactive muscles Practical issues: the weight of limb, vascularity maintenance, internal stabilization of the joined segments override rehabilitation, once those issues are solved a delay to function creates anxiety

Abbreviations: AARoM, active assisted range of movement; ARoM, active range of movement; DIP, distal interphalangeal; MCP, metacarpophalangeal; MRC, Medical Research Council; NMJ, neuromuscular junction; PIP, proximal interphalangeal; PRE, progressive resistance exercise; PRoM, passive range of movement; RUJ, radioulnar joint.

The stages are arranged proximally to the thenar neuromuscular junction (NMJ). Higher-level transplants begin not at stage 2a but lower (i.e., stage 2e). As the recovery occurs, the recipient will arrive at stage 2a. Each phase has a name, the level of surgery, distance to the thenar NMJ, and time to reach it. The latter informs how much time they may spend in that phase. Too much time indicates recovery problems. This should be investigated.

A common question posed by patients is, “When will rehabilitation progress to the next level?” What is being asked for is more purposeful therapies. Initially, they will spend long sessions getting mobilized or having select muscle strengthened. Recipients want to use transplanted hands immediately. Anxiety if they ever will have functional hands emerges. Pre-emptive counseling is relevant. More useful is to set small, specific, time-bound goals. These should be linked to movements that are activities of daily living (ADL) related and in muscles with Medical Research Council (MRC) 3 or more. The recipient can measure their own progress and communicate gains in a productive manner.

Their current function per substage will inform what therapy is needed. Each substage has a goal, which indicates what muscle function should be present with an MRC power of 3 minimum. The goal is linked to a brachial plexus root level. Associated muscles innervated by this root level should be tested and trained for function.

Next is the status of the joints and muscles. This subheading informs status (functional/recovering/status quo/inert) of what is functional from a neuromusculoskeletal perspective. This directly links to the next subheading of interventions. Much of the interventions fall into the domain of standard therapy, which can be delivered by an occupational or physical therapist initially. Eventually, trained family/caregivers should deliver care. To ensure adequate therapy frequency and intensity, involving the family is feasible and optimal. Short, frequent hourly sessions during waking hours are optimal. Each phase intervention has specific caveats. The focus should be to develop extensions at all joints as flexion emerges. Ensure therapists are careful not to overstretch at metacarpophalangeals. This may result in limbs with minimal flexion power. At the end of this stage, orthosis use should be as minimal as possible or discontinued.

### Stages of Aggressive Rehabilitation

**Fig. 2:**
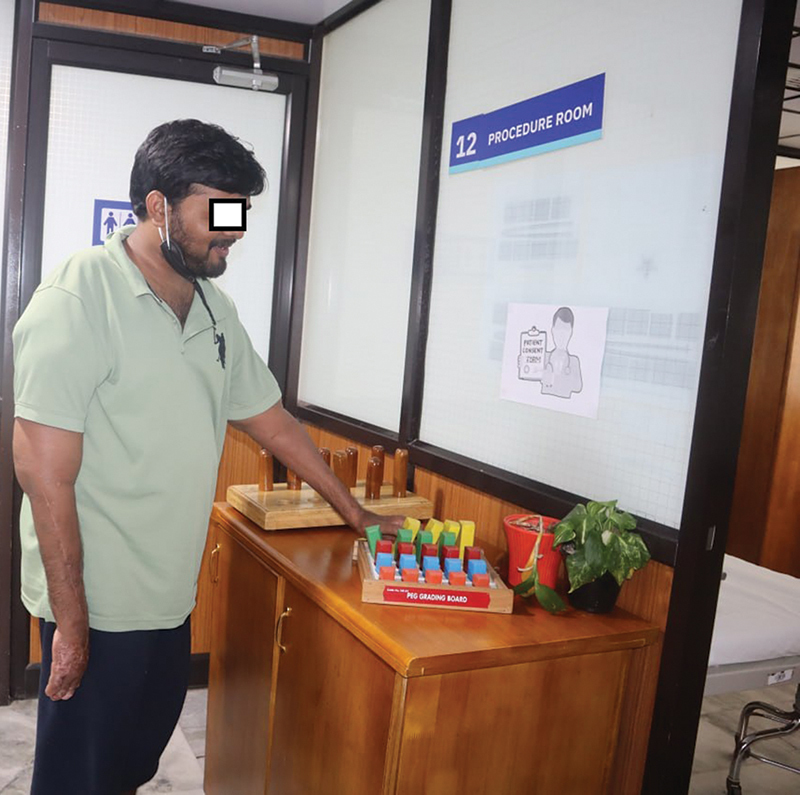
Shoulder level transplant recipient doing occupational therapy.

While they may still require orthoses, the recipient has the capacity for training in ADL. Capacity to perform this is the overarching goal. Once peak performance is achieved, rehabilitation continues from home. There are two phases. The first is the progression of tasks to achieve peak ADL capacity ( Table 3 ). The next is using this to participate as a member of society. Table 4 specifies how to enumerate types of grips. It demarcates progression by RoM, power, and dexterity. Task progression is based on the Quality of Upper Extremity Skills Test (QUEST) score. This can be used to identify specific muscle functions and plan rehabilitation/remediation (i.e., orthoses). They are self-explanatory and require minimal specialized equipment. The lowest row of Table 4 links peak capacity to Chen score. This is a functional outcome measure. On the second column and top row are a series of letter and numbers. As rehabilitation progresses, each grip progression will plateau. These areas are related to the listed tasks in the table. Each task has an alphanumerical code (correlating to the number and letter, respectively). This code indicates ADLs the person should be able to perform ( Table 5 ). This then correlates to activities they can use to participate in society (see Fig. 2 ).

**Table 3:** Summary of stages of aggressive rehabilitation

Phase	Goal		Interventions
3/aggressive intervention	Functional limb training	See functional progression rubric	Occupational therapy to refine motor control using the task progression pattern
4/independent	Reintegration	Vocational retraining by capacity	Use gains to reintegrate into society

**Table 4:** Functional progression rubric

Grasp	Code	A	B	C	D	E	F
Cylinder	1	Hold thick peg	Hold thin peg	Hold pen	Hold pin	X	X
Pinch	2	Fold newspaper	Fold paper	Easy origami	X	X	Hard origami
Three-point chuck	3	Hold bottle top	Unscrew large bottle top	Screw on large bottle top	Unscrew small bottle top	Screw on small bottle top	X
Sphere	4	Grab/catch ball	Throw ball	X	X	X	Baoding balls
Grab	5	Roll dough	X	Cut	X	Tear	X
Fine	6	X	Grab string	Pen and coloring book	Alphabet stencil	Play song on keyboard	String bead/calligraphy
	Chen	1	2	3	4	4	4

**Table 5:** ADL coding

Code	ADL capacity
1C	Eating use utensils, bring food to mouth, chew, and swallow safely
1D	Communication manual tools for speech or writing
2B	Toileting, clothing, cleansing, and hygiene related to toileting
3B	Grooming brushing teeth, washing face, combing hair, shaving, or applying makeup
4A	Bathing and drying trunk and limbs
5A	Bathing and soap application to trunk and limbs
6B	Dressing don/doff shirts, upper undergarments
6B	Dressing donning/doffing pants, lower undergarments

Abbreviation: ADL, activities of daily living.

### Stage of Maintenance

Patients may develop deformities, no recovery of expected functions or deteriorating function. Orthoses or tendon transfers may be needed. Finally, it is the assessment for hand function. This is done 6 and 12 months from the transplant date, and thereafter when and if they come for annual visits. The measures used are passive range of movement, active range of movement, MRC, hand transplantation score system (HTSS), Disabilities of the Arm, Shoulder, and Hand (DASH), grip dynamometer, bulb dynamometers, nonhole peg test, and Semmes–Weinstein Monofilaments.

## Discussion

This is the first rehabilitation protocol for multiple levels of upper extremity transplantation. It is based on the experience from the world's largest cohort of recipients of different levels of hand transplants (34 hands in 18 recipients) from a single center. Existing literature largely focuses on surgery, complications, and outcomes. Sparse publications detail protocols for upper extremity transplant. This work is a rehabilitation framework. The closest similar is a general guideline for a therapist. 15 Posttransplant rehabilitation should be individualized and tailored according to the level of the transplantation to address the concerns in each of these patients. The lack of consensus on outcome measures for assessing hand transplant function, surgical techniques used, patient variability, and various other factors makes it difficult to create a standard universal rehabilitation protocol. Neuromuscular rehabilitation is highly complex, with various factors influencing the outcomes. Physiatrist assessments provide a status overview in relation to the patient's recovery trajectory. They track landmark events. They also troubleshoot the spaces between these, sorting out the need for therapy versus surgical intervention. This is critical to decision-making. If key events do not happen in time, it may warrant further investigation before more therapy happens.

Standard outcome measures are the Purdue Pegboard, Chen score, and the DASH score. They have limitations. Purdue peg board provides an objective metric that is a surrogate marker for function. A small cylinder grasp is required to perform this. This may be challenging in higher-level transplants. Chen score gives overall metric for functional capacity. It does not specify which ADL is impaired, nor why. The DASH is a direct measure of functional capacity in detail for ADL. It is good for pre- and posttreatment assessments. What is missing is a metric that breaks the limb down into components and assesses them functionally. The QUEST was designed for use in cerebral palsy, 16 which fulfils the role.

The longest follow-up is three decades from total hand replant. The article fails to mention rehabilitation details and if the recipient could perform ADL. 17 The oldest known successful hand transplant was done in 1999. No recent publications inform their status. It is known that the recipient is well and independent.

Factors influencing success are occupation, hobbies, belief structures, support system, and motivations. Patients receiving positive reinforcement have better function and mood. 18

Regarding orthoses, the decision-making process was based on the following rationale. Was the goal to support or support with function? 19 In the early phase of nondistal transradial recipients, support is more important than function. Adequate bone and soft tissue healing required before weight bearing is important. The principle of three points of contact remains the basis. As detailed in prior material, choices and frames per level are decided based on the stage and phase. Situation led us to fabricate many custom splints not normally described.

Sensory retraining usually begins when a person has sensation. However, the fact remains that muscles are connected in myofascial chains by their investing fascia. 20 These chains are activated to produce what is seen as functional movements. When muscles are joined in a transplant, Golgi tendon organs/intrafusal fibers of the recipient muscles are active. For example, moving the deltoid or bicep in a proximal level transplant will then activate the same sensors in the named muscles. Feedback to the dorsal horn Rexed lamina (layers five and six) informs the brain how the limb is performing the desired task. As some muscles are not working yet, that is communicated. This input to inadequate output promotes selective reinnervation. For this reason, sensory stimulation is started early. One should note that this is not a standard practice.

The peripheral nervous system does regenerate. Functional recovery after nerve injury and surgical repair may be suboptimal. 21 22 23 Factors such as improper axonal guidance, scar tissue formation, inhibitory factors, delayed repair, slow regeneration, and various other factors can influence the outcomes. When outcomes are not optimal, it is not a failure of regeneration. The distance between the coaptated nerve and the target muscle NMJ influences the time to functional recovery. Achieving reinnervation after denervation is critical. 24 Longer the distance for the nerve to travel, the poorer the recovery of the distal muscles. Higher levels of transplant have higher chances of nil or incomplete recovery distally. Reaching the target NMJ does not ensure functional motor recovery. Disuse apraxia and irreversible changes in the muscles are clinical challenges. 25 The NMJ may remain viable up to 12- to 8 months from deafferentation. It is assumed that electrical stimulation preserves the NMJ. 26 27 Physiatrists must prescribe this with caution. Electric stimulation paradigms fall into two domains: high frequency with low intensity (faradic, used to stimulate nerves) or low frequency with high intensity (interrupted galvanic, used to stimulate muscles). As the nerve is not immediately conducting, the galvanic is used. This may pose danger to the transplanted limbs. Interrupted galvanic voltage may induce burns manifesting as rejection. We recommend using the lowest possible voltage that induces a flicker of contraction. The maximum of 15 contractions per muscle once daily is a safety measure. In our cohort, we noted those with burns related to this stopped receiving it but continued to make gains equal to those who had a similar transplant level and continued receiving it ( Table 6 ).

**Table 6:** Relevant allied health services

Stage	Members
Preparation	Nutrition, psychology
Anticipated movement	Occupational and physical therapy
Aggressive rehabilitation	Occupational therapy
Maintenance	Not required

## Conclusion

A rehabilitation framework specific to each level for the management of hand transplant recipients will lead to optimal outcomes. The rehabilitation regime is better planned and executed if the our stages in rehabilitation are paid attention to. These are the stages of preparation, anticipated movements, and aggressive rehabilitation, and the maintenance phase. Increasing the capacity to do ADL) forms the most important goal, which will allow the patient to go back to an active social life.
